# Medico-Chirurgical Transactions

**Published:** 1847-04

**Authors:** 


### Art. V.

*Medico-Chirurgical Transactions.* Vol. XXVIII.—*London,* 1845. 8vo, pp. 623. Six plates.

We have written so many prefaces to the notices of the preceding volumes of this work, that we can find nothing new to say on the general subject. We shall, therefore, proceed at once to review the contents of the present volume, giving the titles of all the papers, and commenting on such as most call for or best admit of commentary. And it must not be supposed that the papers not commented on do not contain matter of value or importance. All deserve perusal, and several not commented on by us will be found highly interesting.

I. *Case in which the vena cava inferior was obstructed from the commencement of the common iliac veins, and its cavity entirely obliterated between the entrance of the emulgent and hepatic veins ;* by T. B. Peacock, M.D. Physician to the Royal General Dispensary, Aldersgate Street.

II. *On the classification, structure, and development of the echinococcus hominis, showing reasons for regarding it as a species of cysticercus ;* by Erasmus Wilson, F.R.S., Consulting Surgeon to the St. Pancras Infirmary ; Lecturer on Anatomy and Physiology in the Middlesex Hospital.

III. *A case of aneurism of the popliteal artery, cured by compression of the femoral artery ;* by E. Greatrex, Esq. Surgeon, and W. T. C. Robinson. Esq. Assist. Surgeon of the Coldstream Guards.

IV. *On extravasations of blood into the cavity of the arachnoid, and on the formation of the false membrane, which sometimes envelopes these extravasations ;* by Prescott Hewett, Esq. Curator of St. George's Pathological Museum.

The effusions, which are the subject of this last paper, are found in four different states. In the first and simplest, the blood recently extravasated, is found either liquid, or coagulated in the form of clots or of a thin membranous layer, covering a greater or less extent of the surface of the brain. If life be prolonged for any time, this membranous layer becomes partially, or almost altogether deprived of colouring matter, and presents the appearance of a false membrane, which may be found loose in the arachnoid cavity, or, which is by far the most common, be pretty firmly connected with the parietal arachnoid. This is the second state. When thick the membrane is often divisible into distinct layers. Clots of blood, and cysts containing serum and blood, or serum alone, are occasionally met with in the substance of the adventitious tissue. An effusion of this kind has been frequently mistaken for the result of inflammatory action. In the third state, the extravasated blood is fixed to the free surface of the parietal arachnoid by a fine, delicate, transparent membrane, apparently possessing all the characters of the serous membrane itself, of which it at first sight appears to be a part. The blood in these cases, may either be collected in one cavity, or disseminated in patches over the surface of the serous membrane, the intervening parts of which present a natural aspect. In the fourth division, the blood is contained in a completely closed bag, which may be detached from the parietal arachnoid, and with its contents removed unbroken. In the great majority of cases, such encysted collections correspond to the upper surface of the cerebral hemispheres. If the disease be of some standing, the cavity of the cyst may be perfectly smooth and polished, or it may be intersected with fibro-cellular bands, running in various directions; sometimes fibrinous clots are found adhering to this internal surface. After a certain period, the walls of the cyst become fully supplied with blood-vessels, and the cyst itself possesses all the physiological characters of an original serous membrane.

How are these cysts and attaching membranes formed? It is commonly believed that the irritation of the effused blood gives rise to an exudation of lymph; but this explanation, according to Mr. Hewett, is altogether unsatisfactory as regards the majority of cases, seeing that any evidence of inflammation, beyond their presence, is altogether wanting. He believes they are formed by the blood itself; and in support of this view brings forward several examples in which similar effusions, in various localities, have become thus surrounded without the supervention of any inflammatory process.

V. *On the colostrum of the cow;* by John Davy, M.D. F.R.S., Inspector General of Army Hospitals, F.R.S.

VI. *Case of obstruction of the large intestine, in which the ascending colon was opened with success, the patient dying three months afterwards of another disease;* by Samuel Evans, Esq. of Derby.

This is a very interesting case. The advanced stage of the disease and the general condition of the patient rendered success most doubtful; yet the patient recovered perfectly from the effects of the operation, and had regained a good measure of health and strength, when imprudence in diet and exercise brought on an attack of peritonitis, with diabetic urine, which proved fatal. The operation performed was Callisen's modified by Amussat.

VII. *On the mortality in prisons, and the diseases most frequently fatal to prisoners;* by W. Baly, M.D., Physician to the Millbank Prison, and Lecturer on Forensic Medicine at St. Bartholomew's Hospital.

We beg to direct the special attention of our readers to this very valuable communication. In it the author has most conclusively shown, from extensive statistical researches, that the state of imprisonment induces a rate of mortality much higher than that which prevails among the same class of persons unconfined; that this mortality, in some few instances, is the result of certain endemic influences, but that the development of tubercular disease, under various forms, is by far the most efficient operative cause.

VIII. *Observations on cleft palate, and on staphyloraphy;* by W. Fergusson, Esq. Professor of Surgery in King's College, London.

After a few observations on the various forms of cleft palate, and a rapid sketch of the different operations which have been performed for its cure, the author describes the mode of proceeding which he has been led to adopt from a consideration of the anatomy and physiology of the parts. He believes that the chief mechanical obstacles to the junction of the margin in the mesial line, are the levator palati and palato-pharyngeus muscles, and he consequently advises the division of these, by which means all motory power in the soft palate is, for the time being, destroyed. The following is a short account of his operation:

"With a knife whose blade is somewhat like the point of a lancet, the cutting edge being about a quarter of an inch in extent, and the flat surface being bent semicircularly, I make an incision about half an inch long, on each side of the posterior nares, a little above and parallel with the palatine flaps, and across a line straight downwards from the lower opening of the Eustachian tube, by which I divide the levator-palati muscle on both sides, just above its attachment to the palate. Next I pare the edges of the fissure with a straight blunt-pointed bistoury, removing little more than the mucous membrane; then, with a pair of long blunt-pointed scissors, I divide the posterior pillar of the fauces, immediately behind the tonsil, and, if it seems necessary, cut across the anterior pillar too; the wound in each part being about a quarter of an inch in extent. Lastly, the stitches are introduced by means of a curved needle, set in a handle; and, the threads being tied so as to keep the cut edges of the fissure accurately in contact, the operation is completed. These different incisions may be made in the order here detailed, or possibly it may be found most convenient to divide the palato-pharyngeus first, next the levator palati, and then to pare the edges when the muscular action has been taken off."

Two cases are narrated in which the operation was practised with success. The plan seems worthy of full trial, being founded on correct scientific principles.

IX. *On the pulsating tumours of bone, with the account of a case in which a ligature was placed around the common iliac artery;* by Edward Stanley, F.R.S., Surgeon to St. Bartholomew's Hospital.

There are three different sources of pulsation in these tumours. Most commonly the proximity of a large arterial trunk is the cause. Of this condition Mr. Stanley mentions several instances. In more rare cases the pulsation is dependent upon the development of blood-vessels and blood-cells, constituting a sort of erectile tissue within the tumour. The case in which the common iliac was recently tied, at St. Bartholomew's, was of this nature. It originated in the ilium, and was supposed to be an aneurism. The man died from peritonitis on the third day. Still more rarely the pulsation is caused by enlargement of the arteries of the osseous tissue.

The most interesting point in respect of these tumours is the extreme difficulty of diagnosis. They simulate aneurisms so closely that it is sometimes altogether impossible accurately to determine which disease the surgeon has to treat.

X. *Description of a malformation of the duodenum, with notices of analogous cases;* by Robert Boyd, M.D., Resident physician, St. Marylebone Infirmary.

The malformation was discovered in the body of a still-born male infant. The organ was somewhat of an oval shape, six inches long, and two inches in diameter at the lowest and widest part, at the termination of which the canal was completely closed by a transverse membrane. Two inches and a quarter above this a valve extended across, nearly half closing the gut; it proceeded from the concave side, the central attachment of the septum being opposite the mesentery. The stomach was natural. The remainder of the small intestines were very small, and the large intestines were not fully developed. The contents of the cranium were natural.

XI. *Case of remarkable hypertrophy in the fingers in a girl, with a notice of some similar cases;* by T. B. Curling, Lecturer on Surgery, and Assistant Surgeon at the London Hospital, &c.

XII. *On the ophthalmia of puerperal women;* by Robert Lee, M.D. F.R.S., Fellow of the Royal College of Physicians, London; Physician to the British Lying-in Hospital; and Lecturer on Midwifery at St. George's Hospital.

In this paper Dr. Lee brings forward several cases in proof of his opinion that this formidable and rapidly destructive affection of the eyes, is connected with, and consequent upon, uterine phlebitis. His views appear to us important, and consonant with truth.

XIII. *Additional observations on obstructions of the pulmonary arteries;* by James Paget, F.R.C.S., Lecturer on Physiology, and Warden of the College at St. Bartholomew's Hospital.

This is an appendix to a paper in the last vol. of the Transactions. It contains the account of an extremely interesting case, of a man under treatment for stricture of the urethra, who presented no symptoms of pulmonary obstruction, had been only heard to cough once very slightly, and casually mentioned that at times his breath was momentarily rather short. He died suddenly, while in the apparent enjoyment of perfect general health; the fatal event seemingly having been induced by the exertion of defæcation. No disease of any moment was found either in the heart or lungs, but nearly all the branches beyond the primary divisions of the pulmonary artery contained clots of blood, which Mr. Paget conceived to be from three to ten days old. Some very interesting questions are suggested to the mind by such cases as these. How can life be prolonged with so much comfort, while the circulation through such an important organ is so greatly obstructed? Mr. Paget believes this is effected by the proportionally diminished rapidity of the systemic circulation, which follows upon the decreased quantity of blood conveyed to the left ventricle from the lungs. But as the original causes continue in operation, the blood ceases, or nearly ceases, to flow in the systemic vessels; the brain and heart become deprived of their natural stimulus, and sudden or gradual death is the result. But how is the coagulation effected when there is no external cause of obstruction? Mr. Paget is of opinion that it depends upon an altered condition of the blood itself, by which its adhesive properties are augmented, and he points attention to the remarkable fact, that in many of the cases on record, (in the present among others,) the kidneys have been found more or less affected with Bright's disease.

XIV. *Two cases of anæsthesia and loss of motory function of the fifth nerve;* by James Dixon, Esq., Assistant Surgeon to the Royal London Ophthalmic Hospital.

These cases are carefully observed and well narrated, and illustrate some interesting points in physiology.

XV. *Account of a case of external and internal cephalhœmatoma, complicated with fracture of the right parietal bone in a new-born infant;* by Charles West, M.D., Lecturer on Midwifery at the Middlesex Hospital, Physician to the Royal Infirmary for Children, and Physician Accoucheur to the Finsbury Dispensary.

The subject of this case, a female infant, lived for twenty-five days, and then died in convulsions, having exhibited no head symptoms until two days before death. The blood was effused under the pericranium, and between the skull and dura mater; it had evidently been poured out in successive layers. The internal clot was nearly surrounded by a ring of newly-formed bone. There was no reason to believe that the tumour resulted from unnatural violence.

XVI. *Large opening into the anterior part of the urethra, caused by sloughing, and attended by considerable loss of structure, successfully treated by operation, with remarks;* by F. Le Gros Clark, Esq., Assistant Surgeon to St. Thomas's Hospital.

This opening was in the portion of the urethra anterior to the scrotum, and extending a very little way into the scrotal division. Its length, when the penis was turned up, was about 1¼ inch, and the two margins were so far separated as to leave the upper wall of the urethra entirely exposed. The following is Mr. Clark's account of the operation, which is a modification of that proposed by Dieffenbach:

"A large-sized (No. 10) elastic catheter was passed into the bladder, and the stilet being removed, an assistant was directed to hold the penis against the pubes; with a small scalpel I then first removed an inverted portion of skin which bounded the opening posteriorly at the scrotum. I next made, in succession, four incisions through the integument, each commencing at a little distance from the extremity of the urethral deficiency: two of these extended, in contrary directions, upwards and outwards on the sides of the penis, the other two downwards and outwards on the scrotum. Each pair of incisions thus included a nearly rectangular piece of skin between them; and a flap on each side was thus marked out, which grew broader as it extended outwards. I then proceeded (*without* paring the edges, on which I did not at all depend for adhesion) to dissect up these flaps, commencing from the margin of the urethra and advancing outwards, so as to raise the two flaps in question. This being effected, I waited for ten minutes or a quarter of an hour, until the surface-bleeding had been arrested, and then undertook the concluding step of the operation, which was to bring these flaps over the urethra, and apply them *surface to surface*, not edge to edge. For this purpose I had prepared four small quadrilateral pieces of stiff leather, about half an inch square, and perforated. These were applied, two and two, opposite each other, and kept in position by platina wires, which I passed through the leather of each side and the intervening folds of skin, and fastened by twisting the ends together. In this way the flaps were sustained in relation, forming a ridge which projected a little above the pieces of leather, a small intervening portion being left uncompressed. The operation was completed by a semilunar incision on either side of the penis, parallel to the flaps, in order to obviate the effects of any after tension; and three or four sutures were employed at the extremities of the wound."

The cure was eventually complete, and we think the case reflects great credit on the surgeon.

XVII. *The pathology of mental diseases;* by John Webster, M.D. F.R.S., Consulting Physician to St. George's and St. James's Dispensary.

This is a sequel to a paper, which we formerly noticed, in the 26th vol. of the Transactions, and contains the account of a number of further dissections of persons who died insane, which furnished the following general results. The number of dissections was 36. In 33 cases the pia mater was infiltrated. In 30, there was turgidity of the blood-vessels of the brain and its membranes. In 26, effusion of water had taken place in the ventricles. In 16, there was thickening and opacity of the arachnoid. In 12, fluid was met with at the base of the brain. In 9, the consistence of the brain was altered from its normal condition. In 8, patches, or bloody points, appeared on the cut medullary surfaces. In 5, the colour of the medullary, or cortical substance, was altered from its healthy hue to a pink, mottled, or rosy tint; and in 4, blood was effused in the brain. There were also other morbid changes of structure of a less important character.

Dr. Webster gives, likewise, some tables which show that the prevalence of insanity increased with the increased temperature of the season, and that the greatest number of cures took place during the autumnal and winter months.

XVIII. *On some of the causes of pericarditis, especially acute rheumatism and Bright's disease of the kidneys; with incidental observations on the frequency and on some of the causes of various other internal inflammations;* by John Taylor, M.D., Professor of Clinical Medicine in University College, and Physician to University College Hospital.

This is one of those productions that defy analysis. The facts are too numerous for condensation, and too necessary for the argument to allow of omission or abridgment. We have rarely perused any paper that conferred more honour on the writer, and we feel that we are doing an act of simple justice in urging our readers, not merely to go through, but to study its contents. Expanded, and further illustrated, it would form one of the most valuable monographs we possess, and we sincerely hope the author will give it to the profession in this shape. The main object in view is to establish the very important fact, that by far the most frequent causes of pericarditis are two blood-diseases, viz. acute rheumatism and Bright's disease of the kidney. This doctrine is supported by extensive statistical records, and, in our opinion, is conclusively proved. The final conclusions at which the author arrives are thus briefly stated. 1. That acute rheumatism and Bright's disease, *in its advanced stages,* have an *equal* tendency to produce pericarditis and endocarditis. 2. That acute rheumatism has a greater tendency to produce pericarditis and endocarditis than has Bright's disease *when in its earlier stages.* The originality of these views will be at once evident to all. They are worthy of the deepest attention, and open up a wide and most interesting field for future investigations.

XIX. *Case of excision of the upper end of the femur, in an example of morbus coxarius*; by W. Fergusson, esq., Professor of Surgery in King's College, London.

The case terminated most favorably, being only the second of the kind which has had a like result in Britain.

XX. *On the minute structure of the lungs, and on the formation of pulmonary tubercles;* by G. Rainey, esq., M.R.C.S., Microscopist to St. Thomas's Hospital.

The lungs are made up of bronchial tubes, bronchial intercellular passages, and air-cells. The intercellular passages are the continuation of the bronchial tubes, the distinction between the two being the deficiency of the mucous membrane, which terminates abruptly about one eighth of an inch from the surface of the lungs, the remainder of the passage and the air-cells being lined by a very fine, delicate, and distinctly fibrous membrane which has no epithelium. The intercellular passage terminates in an air-cell, which is not dilated, as stated by Reisseissen, but of about the same diameter as the passage. We can bear testimony to the accuracy of this observation.

The air-cells are small, irregular, but generally four-sided, cavities of various sizes, the most central being smallest and most vascular; they communicate freely with each other, and with the intercellular passage. The walls or partitions, by which they are separated from one another, consist of a single plexus of vessels, inclosed in a fold of membrane. The structure, above described, can be seen in the fœtal lung; and, therefore, the cells are not produced, as Mr. Addison describes, by dilatation of the "lobular passages."

According to Mr. Rainey's observations, tubercular matter is poured into the cells from their lining membrane, gradually fills them, compresses the septa, and obliterates the vessels, and thus gradually becomes disintegrated. He denies the inflammatory origin of this morbid product.

XXI. *Two cases of aneurism, in which there was neither pulsation nor abnormal sound;* by T. A. Barker, M.D., Physician to St. Thomas's Hospital.

XXII. *An account of a singular case, in which there was a black secretion from the skin of the forehead and upper part of the face;* by W. Teevan, esq., M.R.C.S.

---

